# Mediterranean Diet Maintained Platelet Count within a Healthy Range and Decreased Thrombocytopenia-Related Mortality Risk: A Randomized Controlled Trial

**DOI:** 10.3390/nu13020559

**Published:** 2021-02-08

**Authors:** Álvaro Hernáez, Camille Lassale, Sara Castro-Barquero, Emilio Ros, Anna Tresserra-Rimbau, Olga Castañer, Xavier Pintó, Zenaida Vázquez-Ruiz, José V. Sorlí, Jordi Salas-Salvadó, José Lapetra, Enrique Gómez-Gracia, Ángel M. Alonso-Gómez, Miquel Fiol, Lluis Serra-Majem, Emilio Sacanella, Cristina Razquin, Dolores Corella, Marta Guasch-Ferré, Montserrat Cofán, Ramón Estruch

**Affiliations:** 1 August Pi i Sunyer Biomedical Research Institute (IDIBAPS), 08036 Barcelona, Spain; sacastro@clinic.cat (S.C.-B.); eros@clinic.cat (E.R.); esacane@clinic.cat (E.S.); MCOFAN@clinic.cat (M.C.); restruch@clinic.cat (R.E.); 2 Consorcio CIBER, M.P. Fisiopatología de la Obesidad y Nutrición (CIBEROBN), Instituto de Salud Carlos III, 28029 Madrid, Spain; classale@imim.es (C.L.); annatresserra@ub.edu (A.T.-R.); ocastaner@imim.es (O.C.); xpinto@bellvitgehospital.cat (X.P.); zvazquez@unav.es (Z.V.-R.); jose.sorli@uv.es (J.V.S.); jordi.salas@urv.cat (J.S.-S.); joselapetra543@gmail.com (J.L.); angelmago13@gmail.com (Á.M.A.-G.); miguel.fiol@ssib.es (M.F.); lserra@dcc.ulpgc.es (L.S.-M.); crazquin@unav.es (C.R.); dolores.corella@uv.es (D.C.); mguasch@hsph.harvard.edu (M.G.-F.); 3 Blanquerna School of Health Sciences, Universitat Ramon Llull, 08025 Barcelona, Spain; 4 Centre for Fertility and Health (CeFH), Norwegian Institute of Public Health, 0473 Oslo, Norway; 5 Cardiovascular Risk and Nutrition Research Group, Hospital del Mar Medical Research Institute (IMIM), 08003 Barcelona, Spain; 6 Department of Medicine, Faculty of Medicine and Health Sciences, University of Barcelona, 08036 Barcelona, Spain; 7 Lipid Clinic, Endocrinology and Nutrition Service, Hospital Clínic, 08036 Barcelona, Spain; 8 Department of Nutrition, Food Science and Gastronomy, XaRTA, INSA, Faculty of Pharmacy and Food Sciences, University of Barcelona, 08028 Barcelona, Spain; 9 Unitat de Nutrició Humana, Departament de Bioquimica i Biotecnologia, Universitat Rovira i Virgili, Hospital Universitari Sant Joan de Reus, 43201 Reus, Spain; 10 Institut d’Investigació Pere Virgili (IISPV), 43204 Reus, Spain; 11 Lipids and Vascular Risk Unit, Internal Medicine Service, Hospital Universitario de Bellvitge, 08907 L’Hospitalet de Llobregat, Spain; 12 Department of Preventive Medicine and Public Health, Universidad de Navarra, 31008 Pamplona, Spain; 13 Department of Preventive Medicine, Universidad de Valencia, 46010 Valencia, Spain; 14 Research Unit, Department of Family Medicine, Distrito Sanitario Atención Primaria Sevilla, 41013 Sevilla, Spain; 15 Department of Preventive Medicine and Public Health, Universidad de Málaga, 29071 Málaga, Spain; egomezgracia@uma.es; 16 Bioaraba Health Research Institute, Osakidetza Basque Health Service, Araba University Hospital, University of the Basque Country UPV/EHU, 01009 Vitoria-Gasteiz, Spain; 17 Health Research Institute of the Balearic Islands (IdISBa), Hospital Son Espases, 07120 Palma de Mallorca, Spain; 18 Instituto de Investigaciones Biomédicas y Sanitarias, Universidad de Las Palmas de Gran Canaria, 35016 Las Palmas, Spain; 19 Centro Hospitalario Universitario Insular Materno Infantil (CHUIMI), Servicio Canario de Salud, 35016 Las Palmas, Spain; 20 Internal Medicine Service, Hospital Clínic, 08036 Barcelona, Spain; 21 Department of Endocrinology and Nutrition, Complejo Hospitalario de Navarra, 31008 Pamplona, Spain; 22 Department of Nutrition, Harvard TH Chan School of Public Health, Boston, MA 02115, USA

**Keywords:** Mediterranean diet, platelet count, thrombocytopenia, randomized controlled trial, prevention

## Abstract

There is little information on the dietary modulation of thrombosis-related risk factors such as platelet count. We aimed to assess the effects of Mediterranean diet (MedDiet) on platelet count and related outcomes in an older population at high cardiovascular risk. In participants of the PREDIMED (PREvención con DIeta MEDiterránea) study, we assessed whether an intervention with a MedDiet enriched with extra-virgin olive oil or nuts, relative to a low-fat control diet, modulated platelet count (*n* = 4189), the risk of developing thrombocytosis and thrombocytopenia (*n* = 3086), and the association between these alterations and all-cause mortality (median follow-up time: 3.0 years). Although platelet count increased over time (+0.98·10^9^ units/L·year [95% confidence interval: 0.12; 1.84]), MedDiet interventions moderated this increase, particularly in individuals with near-high baseline count (both MedDiets combined: −3.20·10^9^ units/L·year [−5.81; −0.59]). Thrombocytopenia incidence was lower in the MedDiet interventions (incidence rates: 2.23% in control diet, 0.91% in MedDiets combined; hazard ratio: 0.44 [0.23; 0.83]). Finally, thrombocytopenia was associated with a higher risk of all-cause mortality (hazard ratio: 4.71 [2.69; 8.24]), but this relationship was attenuated in those allocated to MedDiet (*p*-interaction = 0.018). In brief, MedDiet maintained platelet counts within a healthy range and attenuated platelet-related mortality in older adults at high cardiovascular risk.

## 1. Introduction

Accruing evidence from observational studies and randomized controlled trials such as the PREDIMED (PREvención con DIeta MEDiterránea) study indicates that following a Mediterranean diet (MedDiet) reduces the risk of developing cardiovascular outcomes [1,2]. It is believed that MedDiet may exert these benefits via improving glucose and lipid metabolism, enhancing endothelial function, and decreasing oxidative stress and low-grade inflammation [3]. However, little is known about the effects of MedDiet on thrombosis-related indicators. Following a MedDiet has been associated with improvements in atherothrombosis biomarkers [4], platelet function [5], and decreases in the circulating levels of prothrombotic microvesicles [6]. However, there is little information on the effects of this dietary pattern on other markers related to thrombosis of a more clinical nature, such as platelet count. Platelets are key physiological initiators in clot formation but are increasingly being associated with pathophysiological mechanisms of disease independent of thrombosis [7,8]. Defective and excessive platelet count has been associated with increased mortality and cardiovascular disease incidence in the general population and high cardiovascular risk individuals [9,10,11]. Therefore, finding preventive strategies to avoid the appearance of these alterations seems essential. Following another plant-based dietary pattern (vegan diet) for 4 weeks decreased platelet counts in a small population relative to a meat-rich eating plan (*n* = 53) [12]. High adherence to a MedDiet has only been associated with decreased platelet count in a cross-sectional study [13] and in a small-scale, short-term intervention trial [14]. However, no study has assessed the effects of this dietary pattern on thrombocytosis in the long term and in larger populations, or on other platelet count-related alterations, such as thrombocytopenia, likely to appear in individuals with poor nutritional status [15]. We also do not know whether a possible improvement in platelet count could partially mediate the beneficial effects of MedDiet on chronic diseases and their associated mortality [16].

The aims of our study were to assess in older individuals at high cardiovascular risk whether an intervention with MedDiet (1) maintained platelet count within a healthy range and decreased the risk of developing platelet count-related disorders (thrombocytosis and thrombocytopenia); and (2) modified the association between platelet count-related disorders and all-cause mortality.

## 2. Materials and Methods

### 2.1. Study Population

The study population consisted of participants of the PREDIMED study, which was a multicenter, randomized, controlled trial conducted in Spain between 2003 and 2010 assessing the effects of following a MedDiet on the primary prevention of cardiovascular outcomes in an older population at high cardiovascular risk. Eligible participants were men (aged 55–80 years) and women (aged 60–80 years) free of cardiovascular disease at enrolment but with type-2 diabetes or three or more of the following risk factors: (1) smoking; (2) hypertension; (3) high concentrations of low-density lipoprotein cholesterol; (4) low levels of high-density lipoprotein cholesterol; (5) overweight/obesity; and (6) family history of premature coronary heart disease. Enrolment commenced on 25 June 2003, and the last participant was included on 30 June 2009. The PREDIMED study was registered under the International Standard Randomized Controlled Trial Number ISRCTN35739639 (http://www.isrctn.com/ISRCTN35739639 (accessed on 8 January 2021)). The study protocol complied with the Declaration of Helsinki and was endorsed by institutional review boards of all recruiting centers. An institutional ethics committee (CEIC-PSMAR) approved the particular protocol of this sub-project (code: 2018/8180/I; date: 4 December 2018). All volunteers provided written informed consent before entering the trial. The protocol, recruiting methods, and data collection processes have been described in previous publications [1,17] and are available in the PREDIMED study website (http://www.predimed.es (accessed on 8 January 2021)).

**Figure 1:**
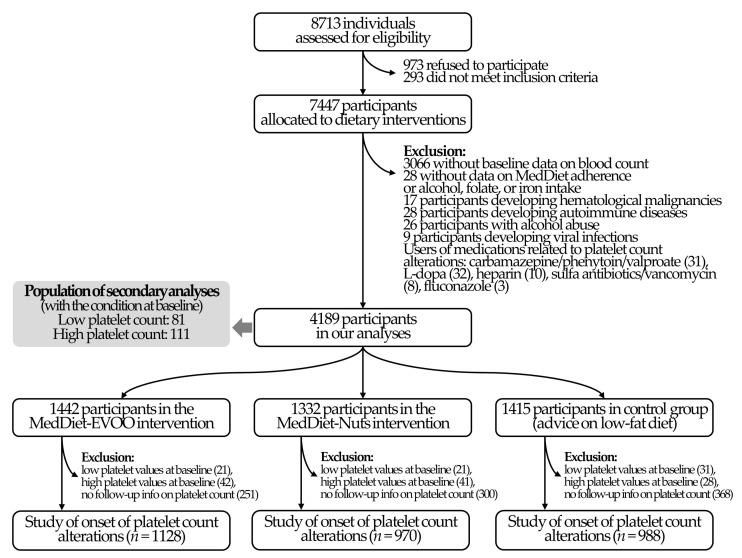
Study flowchart. MedDiet: Mediterranean diet; MedDiet-EVOO: MedDiet enriched with extra-virgin olive oil; MedDiet-Nuts: MedDiet enriched with mixed nuts.

Platelet count alterations were not a predefined endpoint in the PREDIMED study, and thus this study should be considered as exploratory. Of the 4381 PREDIMED participants with blood count available, we excluded 28 individuals without baseline data on MedDiet adherence or intake of alcohol, folate, and iron. To exclusively determine the effects on platelet levels of the dietary intervention, we also excluded those participants with any condition potentially related to alterations in platelet count at any point of the study [15,18], including the following: (1) any cancer of the immune system (17 individuals); (2) an autoimmune disease (28 individuals, determined as the use of immunosuppressant medications); (3) alcohol abuse (26 individuals, determined as a cumulative average of alcohol intake throughout the study of ≥4 drinks/day in men or ≥3 drinks/day in women); (4) viral infections (9 participants, determined as the use of oral antiviral medication); and (5) users of medications associated with platelet count alterations (10 users of heparins, 31 users of certain anticonvulsants (carbamazepine, phenytoin, and valproate), 32 users of L-dopa, 8 users of certain antibiotics (sulfamethoxazole, sulfa antibiotics, and vancomycin), and 3 fluconazole users). No participants with health outcomes related to altered platelet count (aplastic anemia, myelodysplastic syndromes, paroxysmal nocturnal hemoglobinuria, thrombotic thrombocytopenic purpura, disseminated intravascular coagulation syndrome, hemolytic uremic syndrome, Wiskott–Aldrich syndrome, May–Hegglin anomaly, etc.) were included in the trial. This yielded a main analytical sample of 4189 participants. For the analyses on the risk of developing platelet count-related alterations, we additionally excluded those individuals with thrombocytopenia or thrombocytosis at baseline (81 and 111, respectively) and those without information of platelet count in follow-up visits (911 participants) (sample size: 3086 individuals). The flowchart of the study is available in Figure 1. 

### 2.2. Dietary Intervention

Three intervention arms (to which volunteers were randomly allocated according to a 1:1:1 ratio) were compared: (1) a MedDiet enriched with extra-virgin olive oil (MedDiet-EVOO); (2) a MedDiet enriched with mixed nuts (MedDiet-Nuts); and (3) a low-fat control diet. MedDiet interventions encouraged (1) the intake of vegetables, fruits, pulses, nuts, and fish; (2) the use of extra-virgin olive oil as the main culinary fat; (3) a reduction in the consumption of sugary drinks, commercial bakery goods, pastries, sweets, and spread fats; (4) the replacement of red/processed meats for poultry; and (5) food preparation following home-made recipes (such as the traditional “sofrito”, tomato-based stir-fried sauce with olive oil, onion, and garlic). To promote compliance and account for family needs, volunteers allocated in the MedDiet-EVOO intervention received 1 L/week of extra-virgin olive oil and those in the MedDiet-Nuts group were provided with 210 g/week of mixed nuts. Volunteers allocated to the low-fat control group were advised (1) to increase the consumption of vegetables, fruits, pulses, low-fat dairy products, and lean fish; and (2) to reduce their intake of vegetable oils (including olive oil), commercial bakery goods and sweets, nuts and fried snacks, fatty fish, seafood canned in oil, red and processed fatty meats, visible fat in meats and soups, spread fats, and “sofrito”. Additional details of the dietary protocol are available elsewhere [1,17].

### 2.3. Outcomes

Platelet count was measured at baseline in all 4189 participants in the main analytical sample and at least in one study follow-up point in 3086 individuals in fasting blood samples by automated analyzers [19]. The normal range in an older Mediterranean population is 122 to 350 × 10^9^ platelets/L in men and 140 to 379 × 10^9^ platelets/L in women [20]. Thus, thrombocytopenia (low platelet count) was defined as presenting <122 in men and <140·10^9^ units/L in women, and thrombocytosis (high platelet count) was defined as presenting >350 in men and >379·10^9^ units/L in women. We calculated incidence and time-to-event of the onset of any of these alterations among volunteers with platelet counts within the physiological range at study baseline. We defined “onset” as the occurrence of any of the platelet count alterations in one of the follow-up visits that persisted until the last visit for which data is available. We considered a valid onset as any occurrence that persisted for at least three subsequent follow-up visits and presented no more than one “return to normal” value.

For our second aim, we collected information on all-cause mortality. Occurrence up to 1 December 2010 and time-to-event values were determined by the Clinical Event Committee through follow-up study visits, yearly review of medical records, repeated contact with the participants, and linkage with the national death registry [1,17].

### 2.4. Covariates

Trained personnel collected data on the following variables in the baseline visit: age; sex; educational level; prevalence of diabetes, hypercholesterolemia, hypertriglyceridemia, and hypertension; use of antiplatelet drugs; body mass index; and smoking habit. To account for any indicators related to low-grade inflammation, white blood cell counts were measured in fasting blood samples in automated analyzers, as previously described [19]. We estimated physical activity levels in metabolic equivalents of task-minute per day from the Minnesota Leisure-Time Physical Activity Questionnaire validated in the Spanish population. Finally, we estimated the intake of alcohol (in g/day), dietary folate (in μg/day), and iron (in mg/day) from a validated 137-item food frequency questionnaire [1,17].

### 2.5. Power Analyses

The number of total individuals and cases that occurred during follow-up in each study group allowed ≥80% power to detect as significant (*p*-value < 0.05) hazard ratios (HR) lower than 0.42, 0.35, and 0.33 (for the comparisons of both MedDiets combined, MedDiet-EVOO, and MedDiet-Nuts, with control diet, respectively) in relation to the onset of thrombocytopenia, and lower than 0.48, 0.40, and 0.35 regarding the onset of thrombocytosis (Supplementary Materials Table S1). We performed these analyses using the “powerSurvEpi” package in R Software [21].

### 2.6. Statistical Analyses

We described the characteristics of the participants at baseline by means and standard deviation (normally distributed continuous variables), medians and interquartile range (non-normally distributed continuous variables), and proportions (categorical variables).

We studied the effects of MedDiet interventions on platelet count evolution by repeated measurement mixed linear models. We assessed time effects (continuous yearly change across the overall study population) and between-group changes (difference in changes over time in the MedDiet intervention arms—individually and combined—relative to the control diet group). We performed the previous analyses in the overall study population, as well as stratifying participants in quartiles according to platelet count at baseline (after testing whether there was a significant interaction between baseline platelet count and intervention group, by applying a likelihood ratio test between the nested models with and without the interaction product term). Estimates were adjusted for age (continuous), sex, educational level (primary, secondary, greater, unavailable information), recruitment site, diabetes (yes/no), hypercholesterolemia (yes/no), hypertriglyceridemia (yes/no), hypertension (yes/no), use of antiplatelet drugs (yes/no), smoking habit (current/former/never smoker), white blood cell count (continuous), leisure-time physical activity (continuous), body mass index (continuous), alcohol consumption (continuous), folate intake (continuous), iron consumption (continuous), and two propensity scores that used 30 baseline variables to estimate the probability of assignment to each of the intervention groups [1]. We fitted models using the “lme4” package in R Software (Vienna, Austria) [22].

We assessed the differences in the risk of developing thrombocytopenia and thrombocytosis using two sets of Cox proportional hazards regression models. We defined follow-up time as the time between the date of enrolment and (1) the midpoint between the last visit without the outcome and the first visit in which the alteration was registered [23]; (2) 5 years of maximum follow-up time; or (3) 1 December 2010, whichever came first. Any onset of platelet count alteration after a diagnosis of cancer was not considered a valid outcome (3 thrombocytopenias, 3 thrombocytosis). We evaluated whether there were differences in the risk of presenting the outcome in the MedDiet intervention groups (individually and combined) relative to the control diet and fitted two models. Model 1 was stratified by sex and recruitment site and adjusted for age. Model 2 was further stratified by educational level and adjusted for baseline platelet count and the rest of the covariates described in the repeated measurement analyses. We used robust variance estimators to minimize intra-cluster correlations [1] and fitted models using the “survival” package in R Software [24]. We also represented Kaplan–Meier cumulative incidence curves for each study group, weighted by inverse probability weighting with a propensity score model of assignment to intervention or control group based on the covariates above listed.

Finally, we determined whether MedDiet modified the association between platelet count alterations at baseline and all-cause mortality. We compared the volunteers allocated to MedDiet intervention relative to those in the control group. We fitted Cox models where the outcome was major adverse cardiovascular events or all-cause mortality, included an interaction product term of “platelet count alteration × intervention group”, and applied a likelihood ratio test between the nested models with and without it.

We performed all analyses with R Software, version 3.5.2 [25].

## 3. Results

### 3.1. Study Population

Our participants were older adults (67 years old on average, 58% women) with a high prevalence of cardiovascular risk factors (84% hypertension, 74% hypercholesterolemia, 49% obesity, 47% diabetes, 30% hypertriglyceridemia, 13% current smokers) (Table 1), followed for a median time of 3.0 years.

### 3.2. MedDiet Interventions and Platelet Count

Overall platelet count increased over the follow-up in the whole study population (+0.98·10^9^ units/L·year [95% confidence interval: 0.12; 1.84]). However, the MedDiet-Nuts intervention moderated this increase throughout the study (−1.21·10^9^ units/L·year [−2.39; −0.027], less increase than the control low-fat group). Intervention effects depended on baseline platelet levels (*p*-value for interaction with both MedDiets combined = 0.020; *p*-value for interaction with MedDiet−EVOO = 0.080; *p*-value for with MedDiet−Nuts = 0.019). In stratified analysis by platelet count at baseline, both MedDiet interventions combined (compared to the low-fat control group) particularly contributed to maintaining the platelet count within a normal range in subjects with near-high baseline counts, namely the fourth quartile (−3.20·10^9^ units/L·year [−5.81; −0.59]). The MedDiet-Nuts intervention induced this effect in subjects with baseline levels in the third (−2.17·10^9^ units/L·year [−4.13; −0.21]) and fourth quartile (−4.13·10^9^ units/L·year [−7.17; −1.09]), whilst a similar outcome was also suggested in the MedDiet-EVOO group in individuals in the fourth quartile (−2.48·10^9^ units/L·year [−5.36; 0.40]) (Table 2).

### 3.3. MedDiet Interventions and Platelet Count-Related Disorders

As observed in Table 3, both MedDiet interventions were associated with a decreased risk of developing thrombocytopenia. Incidence rates were 2.23% in the control group, 0.91% in both MedDiets combined, 0.89% in the MedDiet-EVOO, and 0.93% in the MedDiet-Nuts. Both MedDiet interventions together decreased the risk of thrombocytopenia by 56% (HR: 0.44 [0.23; 0.83]) compared to the control group. When analyzing MedDiet arms individually, the MedDiet-EVOO was also associated with less risk (HR: 0.36 [0.16; 0.80]), whilst the MedDiet-Nuts was linked to lower risk in the model only adjusted for age, sex, and recruitment site (HR: 0.40 [0.18; 0.89]). There was no evidence of an effect on the risk of developing thrombocytosis. Weighted Kaplan–Meier curves are available in Supplementary Materials Figure S1.

### 3.4. Interaction between Platelet Count-Related Disorders at Baseline and MedDiet on All-Cause Mortality

**Figure 2:**
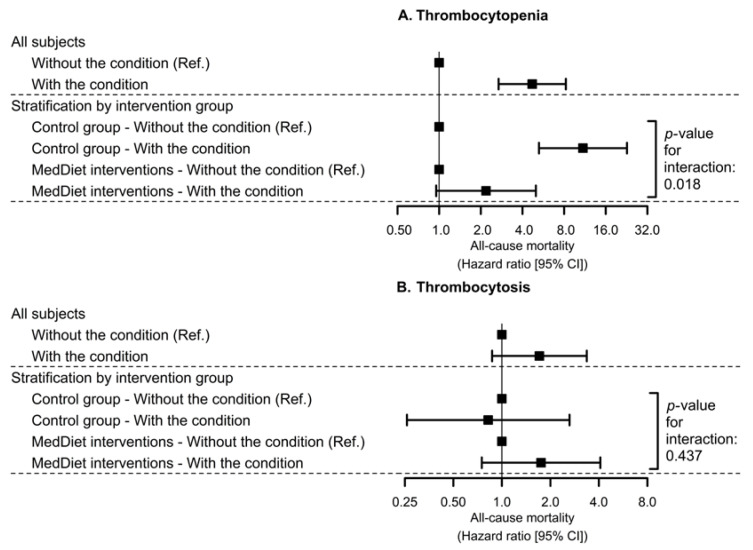
Associations of thrombocytopenia (**A**) and thrombocytosis (**B**) at baseline with all-cause mortality risk stratified by MedDiet groups. Hazard ratios were estimated by multivariable Cox proportional hazards regression models stratified by sex, recruitment site, and educational level and adjusted for age, baseline platelet count, diabetes, hypercholesterolemia, hypertriglyceridemia, hypertension, antiplatelet drug use, smoking habit, white blood cells, leisure-time physical activity, body mass index, intake of alcohol, folates, and iron (at baseline, all), as well as two propensity scores that used 30 baseline variables to estimate the probability of assignment to each of the intervention groups. We used robust standard errors to account for intra-cluster correlations. MedDiet: Mediterranean diet.

Thrombocytopenia was robustly associated with a higher risk of all-cause mortality (HR: 4.71 [2.69; 8.24]). This relationship was stronger for participants in the control diet group and blunted in those allocated to the MedDiet intervention groups (HR_control diet_: 10.9 [5.26; 22.8]; HR_MedDiet_: 2.18 [0.95; 5.00]; *p*-interaction: 0.018) (Figure 2A). No interaction between thrombocytosis at baseline and group allocation on all-cause mortality was found (Figure 2B). Specific values are available in Supplementary Materials Table S2.

## 4. Discussion

Our findings suggest that MedDiet contributed to maintaining platelet counts within the normal range in an older population at high cardiovascular risk. In addition, MedDiet interventions also decreased the risk of developing thrombocytopenia and attenuated the association of this platelet count-related alteration with all-cause mortality.

Low and high platelet counts are risk factors for increased mortality and cardiovascular incidence in the general population and high cardiovascular risk individuals [9,10,11]. In the particular case of high platelet count, it has been associated with greater cardiovascular disease and cancer incidence and mortality [10,26]. The association of platelet count with cardiovascular disease and some types of cancer (lung) has been proven to be causal in some Mendelian randomization studies [27,28]. Therefore, reducing platelets via lifestyle intervention could have high therapeutic value. Our findings suggest that platelet count tends to increase over time and show that following a MedDiet contributes to maintaining platelet counts within normal levels in an older population at high cardiovascular risk. This last effect was particularly marked in individuals starting off with near-high platelet count values, who are likely to benefit from a decrease [26]. In view of our findings, we could expect that platelet counts in MedDiet groups could be 16·10^9^ counts/L lower when compared to the control diet at the end of the study follow-up, although this decrease seemed insufficient to decrease the risk of developing thrombocytosis. An increase in the genetically predetermined levels of platelets of 51.1·10^9^ platelets/L in men and 55.3·10^9^ platelets/L in women was associated with a 7% increase in the risk of ischemic stroke and a 12% increment in risk of cardioembolic stroke independently from the baseline platelet count [27]. Assuming a linear, continuous association between platelet levels and outcome risk, a reduction in platelet count similar to the one observed in our study would be linked to a 2.0–2.2% decrease in ischemic stroke risk and a 3.5–3.8% decline in cardioembolic stroke risk, a modest reduction in the risk of cerebrovascular event but within the magnitude expected for a realistic lifestyle modification. An association between high adherence to a MedDiet and reduced platelet count was reported in a cross-sectional study [13] and in a small-scale, short-term intervention trial [14], and another plant-based dietary pattern (vegan diet) was shown to be able to moderate this parameter in the short term in a recent small-sized study [12]. However, this is the first time that a long-term effect has been reported in a large randomized controlled trial with repeated measurements. The capacity of the MedDiet to decrease inflammation could help with explaining this effect [29,30], given the close relationship between inflammatory status and platelet levels [31].

Low platelet count states are associated with a greater risk of infectious disease and mortality in cohort studies on the general population [9], in individuals at high cardiovascular risk [32], and in acute conditions, such as COVID-19 infection [33]. Beyond the maintenance of platelet count within normal ranges, MedDiet interventions appeared to lower the risk of developing thrombocytopenia in older adults at high cardiovascular risk, and attenuated all-cause mortality risk among individuals presenting the condition. Deficient levels of platelets and their consequences are traditionally addressed by pharmacological treatments [34]. However, to the best of our knowledge, this is the first intervention study to date to describe the protective effect of a healthy diet on the development of low platelet count and their associated mortality. Our main hypothesis to explain the decrease in the risk of developing thrombocytopenia and some of its potential complications was the improvement in the general nutritional status of the participants in the MedDiet intervention groups, which may subsequently lead to an improved production of platelets in those participants at risk of presenting low platelet count, since platelet production and thrombocytopenia risk appear to be highly dependent on the nutritional status of the individuals [15].

Our study presents some limitations. First, platelet count alterations were not a predefined endpoint in the PREDIMED study and, therefore, our findings should be considered as exploratory. Second, our participants were investigated between 2003 and 2009, and thus it would be advisable that our findings should be re-evaluated in more recent studies. Third, we cannot generalize our findings to other populations than older adults with a high prevalence of cardiovascular risk factors. Fourth, platelet count alterations were defined according to threshold values obtained in Mediterranean populations and had a low incidence in our study. Therefore, our results should be interpreted with caution. Fifth, platelet levels can present short-term fluctuations, but we overcame this limitation by using repeated measures. Sixth, considering that MedDiet interventions were modest real-life dietary modifications and that the control diet was a healthy dietary pattern, only moderate effects on platelet-related outcomes could be reported. Finally, residual confounding may have resulted from self-reported information on leisure-time physical activity and the consumption of alcohol, dietary folate, and iron.

## 5. Conclusions

In conclusion, following a MedDiet intervention contributed to maintaining platelet counts within normal ranges, a surrogate risk factor for several chronic diseases that tended to increase over time. This effect was particularly strong in participants presenting near-high platelet counts at baseline. In parallel, the MedDiet intervention also decreased the risk of developing thrombocytopenia and attenuated the association of this platelet count alteration with all-cause mortality. Despite the limitation of the small number of subjects in some subgroups, to the best of our knowledge, this is the first intervention study reporting beneficial effects of a healthy dietary pattern on thrombocytopenia and its related disorders. In parallel, our findings also confirm the long-term capacity of MedDiet to moderate the increase in platelet count over time. Our results suggest the capacity of MedDiet to keep platelet count in its typical range, which may have a great clinical impact, since both low and high platelet counts are risk factors for several lethal outcomes.

## Figures and Tables

**Table 1:** Study population ^1^.

	All Participants	MedDiet-EVOO	MedDiet-Nuts	Control Diet
	(*n* = 4189)	(*n* = 1442)	(*n* = 1332)	(*n* = 1415)
Age (years), mean ± *SD*	67.0 ± 6.14	66.7 ± 6.06	66.9 ± 6.04	67.5 ± 6.30
Female sex, *n* (%)	2430 (58.0)	859 (59.6)	725 (54.4)	846 (59.8)
Diabetes, *n* (%)	1954 (46.6)	686 (47.6)	611 (45.9)	657 (46.4)
Hypercholesterolemia, *n* (%)	3100 (74.0)	1069 (74.1)	996 (74.8)	1035 (73.1)
Hypertriglyceridemia, *n* (%)	1264 (30.2)	439 (30.4)	405 (30.4)	420 (29.7)
Hypertension, *n* (%)	3522 (84.1)	1201 (83.3)	1121 (84.2)	1200 (84.8)
Antiplatelet users, *n* (%)	830 (19.8)	255 (17.7)	284 (21.3)	291 (20.6)
Smoking habit:				
Never smokers, *n* (%)	2589 (61.8)	895 (62.1)	798 (59.9)	896 (63.3)
Current smokers, *n* (%)	562 (13.4)	195 (13.5)	186 (14.0)	181 (12.8)
Former smokers, *n* (%)	1038 (24.8)	352 (24.4)	348 (26.1)	338 (23.9)
Weight (according to body mass index):				
18.5–24.9 kg/m^2^, *n* (%)	281 (6.71)	100 (6.93)	101 (7.58)	80 (5.65)
25.0–29.9 kg/m^2^, *n* (%)	1860 (44.4)	645 (44.7)	610 (45.8)	605 (42.8)
≥30.0 kg/m^2^, *n* (%)	2048 (48.9)	697 (48.3)	621 (46.6)	730 (51.6)
Leisure-time physical activity (metabolic equivalents of task-min/day), median (1st–3rd quartile)	166 (56.1–312)	175 (60.8–319)	182 (63.3–326)	150 (46.4–280)

^1^ MedDiet-EVOO: Mediterranean diet intervention enriched with extra-virgin olive oil; MedDiet-Nuts: Mediterranean diet intervention enriched with mixed nuts.

**Table 2:** Time-dependent changes in platelet count in the PREDIMED study groups ^1^.

	Baseline	1 Year	2–3 Years	4–5 Years	Time Effect	Time × Group Effect
	Mean ± *SD*	Mean ± *SD*	Mean ± *SD*	Mean ± *SD*	(10^9^ Units/L·Year, [95% CI])	(10^9^ Units/L·Year, [95% CI], vs. Control Diet)
All individuals						
Control diet	235 ± 60.4	236 ± 60.7	237 ± 64.1	238 ± 64.2	+0.98	Ref.
MedDiet combined	235 ± 60.5	237 ± 61.9	236 ± 60.8	236 ± 65.6	[0.12; 1.84]	−0.82 [−1.83; 0.19]
MedDiet-EVOO	236 ± 60.1	236 ± 62.7	239 ± 61.9	239 ± 67.7		−0.52 [−1.64; 0.60]
MedDiet-Nuts	235 ± 61.1	237 ± 61.1	234 ± 59.2	232 ± 62.4		−1.21 [−2.39; −0.027]
1st quartile (<194·10^9^ platelets/L at baseline)						
Control diet	166 ± 24.0	180 ± 40.3	176 ± 42.0	185 ± 42.6	+3.67	Ref.
MedDiet combined	168 ± 23.8	180 ± 41.8	180 ± 36.6	183 ± 43.4	[2.30; 5.04]	−0.024 [−1.65; 1.60]
MedDiet-EVOO	167 ± 24.2	180 ± 37.9	181 ± 34.2	182 ± 41.6		−0.056 [−1.87; 1.76]
MedDiet-Nuts	168 ± 23.5	180 ± 46.2	180 ± 39.4	184 ± 45.9		0.032 [−1.87; 1.93]
2nd quartile (194–229·10^9^ platelets/L at baseline)						
Control diet	212 ± 9.71	217 ± 31.1	217 ± 34.0	221 ± 35.0	+1.41	Ref.
MedDiet combined	212 ± 9.96	219 ± 36.1	216 ± 39.4	218 ± 38.8	[0.12; 2.70]	−0.33 [−1.84; 1.18]
MedDiet-EVOO	213 ± 9.65	218 ± 35.0	218 ± 43.6	218 ± 39.4		−0.42 [−2.10; 1.26]
MedDiet-Nuts	211 ± 10.3	220 ± 37.3	214 ± 32.4	218 ± 38.2		−0.22 [−1.99; 1.55]
3rd quartile (229–268·10^9^ platelets/L at baseline)						
Control diet	248 ± 10.9	248 ± 33.3	245 ± 34.6	252 ± 40.3	+0.86	Ref.
MedDiet combined	248 ± 11.0	247 ± 33.2	246 ± 34.6	252 ± 48.9	[−0.56; 2.28]	−0.43 [−2.10; 1.24]
MedDiet-EVOO	249 ± 11.1	247 ± 32.3	250 ± 35.3	256 ± 43.7		0.85 [−1.00; 2.70]
MedDiet-Nuts	248 ± 10.9	247 ± 34.1	241 ± 33.3	246 ± 54.7		−2.17 [−4.13; −0.21]
4th quartile (≥268·10^9^ platelets/L at baseline)						
Control diet	315 ± 44.5	300 ± 54.9	305 ± 56.6	318 ± 59.4	−1.46	Ref.
MedDiet combined	316 ± 45.3	301 ± 60.7	297 ± 57.1	299 ± 67.7	[−3.70; 0.78]	−3.20 [−5.81; −0.59]
MedDiet-EVOO	316 ± 43.2	303 ± 65.5	299 ± 59.2	306 ± 70.9		−2.48 [−5.36; 0.40]
MedDiet-Nuts	317 ± 47.6	299 ± 55.2	294 ± 54.3	288 ± 61.6		−4.13 [−7.17; −1.09]

^1^ Time effects and between-group changes were estimated by repeated measurement mixed models adjusted for age, sex, educational level, recruitment site, diabetes, hypercholesterolemia, hypertriglyceridemia, hypertension, antiplatelet drug use, smoking habit, white blood cells, leisure-time physical activity, body mass index, intake of alcohol, folates, and iron (at baseline, all), as well as two propensity scores that used 30 baseline variables to estimate the probability of assignment to each of the intervention groups. MedDiet-EVOO: Mediterranean diet intervention enriched with extra-virgin olive oil; MedDiet-Nuts: Mediterranean diet intervention enriched with mixed nuts.

**Table 3:** Incidence of platelet count-related alterations in the study population ^1^.

	Thrombocytopenia			Thrombocytosis		
	Cases/Total	Model 1	Model 2	Cases/Total	Model 1	Model 2
	(Incidence Rate)	HR [95% CI]	HR [95% CI]	(Incidence Rate)	HR [95% CI]	HR [95% CI]
Control diet	22/988			18/988		
	(2.23%)	1 (Ref.)	1 (Ref.)	(1.82%)	1 (Ref.)	1 (Ref.)
MedDiets combined	19/2098	0.34	0.44	40/2098	0.96	1.29
	(0.91%)	[0.18; 0.64]	[0.23; 0.83]	(1.91%)	[0.55; 1.69]	[0.74; 2.26]
MedDiet-EVOO	10/1128	0.29	0.36	24/1128	1.04	1.57
	(0.89%)	[0.14; 0.62]	[0.16; 0.80]	(2.13%)	[0.56; 1.93]	[0.84; 2.97]
MedDiet-Nuts	9/970	0.40	0.56	16/970	0.86	1.04
	(0.93%)	[0.18; 0.89]	[0.26; 1.21]	(1.65%)	[0.44; 1.69]	[0.52; 2.06]

^1^ Thrombocytopenia has been defined as the presence of <122 × 10^9^ platelets/L in men and <140 × 10^9^ platelets/L in women, and thrombocytosis has been defined as the presence of >350 × 10^9^ platelets/L in men and >379 × 10^9^ platelets/L in women. Hazard ratios were estimated by multivariable Cox proportional hazards regression models. Model 1 was stratified by sex and recruitment site and adjusted for age. Model 2 was further stratified by educational level and adjusted for baseline platelet count, diabetes, hypercholesterolemia, hypertriglyceridemia, hypertension, antiplatelet drug use, smoking habit, white blood cells, leisure-time physical activity, body mass index, intake of alcohol, folates, and iron (at baseline, all), as well as two propensity scores that used 30 baseline variables to estimate the probability of assignment to each of the intervention groups. We used robust standard errors to account for intra-cluster correlations. HR: hazard ratio; MedDiet-EVOO: Mediterranean diet intervention enriched with extra-virgin olive oil; MedDiet-Nuts: Mediterranean diet intervention enriched with mixed nuts.

## Data Availability

The dataset analyzed during the current study is not publicly available due to national data regulations and for ethical reasons, including that we do not have the explicit written consent of the study volunteers to make their deidentified data available at the end of the study. However, datasets and R codes of data management/transformation and statistical analyses can be requested by sending a letter to the PREDIMED Steering Committee (predimed-steering-committe@googlegroups.com). The request will then be passed to all the members of the committee for deliberation.

## Supplementary Materials

The following are available online at https://www.mdpi.com/2072-6643/13/2/559/s1: Figure S1: Weighted Kaplan–Meier estimates of the cumulative incidence of thrombocytopenia and thrombocytosis in intervention groups; Table S1: Power analyses; Table S2: Associations of platelet count alterations at baseline with the risk of all-cause mortality stratified by Mediterranean diet intervention group.
