# Supplementary material for: Mediterranean Diet Maintained Platelet Count within a Healthy Range and Decreased Thrombocytopenia-Related Mortality Risk: A Randomized Controlled Trial
Source: Nutrients. 2021 Feb 8;13(2):559. doi: 10.3390/nu13020559 (PMC7915168; doi:10.3390/nu13020559)
Supplement: Supplementary file 1 [file nutrients-13-00559-s001.pdf]

SUPPLEMENTAL MATERIALS

SUPPLEMENTAL TABLES

Supplemental Table 1. Power analyses

	Volunteers (control diet)	**MedDiets combined vs. control diet**				**MedDiet-EVOO vs. control diet**				**MedDiet-Nuts vs. control diet**			
		Volunteers (MedDiet-EVOO)	Cases (control + MedDiet-EVOO)	HR detectable with ≥80% power		Volunteers (MedDiet-Nuts)	Cases (control + MedDiet-Nuts)	HR detectable with ≥80% power		Volunteers (MedDiets combined)	Cases (control + MedDiets combined)	HR detectable with ≥80% power	
				<1	>1			<1	>1			<1	>1
Onset of thrombocytopenia	988	2,098	41	≤0.42	≥3.55	1,128	32	≤0.35	≥3.09	970	31	≤0.33	≥2.91
Onset of thrombocytosis	988	2,098	58	≤0.48	≥2.68	1,128	42	≤0.40	≥2.60	970	34	≤0.40	≥2.82

HR: hazard ratio; *MedDiet-EVOO*: Mediterranean diet enriched with extra-virgin olive oil; *MedDiet-Nuts*: Mediterranean diet enriched with mixed nuts.

Supplemental Table 2. Associations of platelet count alterations at baseline with the risk of all-cause mortality stratified by Mediterranean diet intervention group.

		Thrombocytopenia			Thrombocytosis		
Association of platelet count alterations (at baseline) with all-cause mortality							
	Presence of the alteration	Cases/Total	HR [95% CI]	*P*-value	Cases/Total	HR [95% CI]	*P*-value
	No	154/3,997 (3.85%)	1 (Ref.)		154/3,997 (3.85%)	1 (Ref.)	
	Yes	16/81 (19.8%)	4.71 [2.69; 8.24]	<0.001	10/111 (9.01%)	1.71 [0.87; 3.37]	0.120
Stratification in PREDIMED intervention groups							
	Presence of the alteration	Cases/Total	HR [95% CI]	Interaction (*P*-value)	Cases/Total	HR [95% CI]	Interaction (*P*-value)
Control diet	No	47/1,360 (3.46%)	1 (Ref.)		47/1,360 (3.46%)	1 (Ref.)	
	Yes	9/27 (33.3%)	10.9 [5.26; 22.8]		2/28 (7.14%)	0.82 [0.26; 2.63]	
MedDiet groups	No	107/2,637 (4.06%)	1 (Ref.)		107/2,637 (4.06%)	1 (Ref.)	
	Yes	7/54 (13.0%)	2.18 [0.95; 5.00]	0.018	8/83 (9.64%)	1.75 [0.75; 4.08]	0.437

Hazard ratios were estimated by multivariable Cox proportional hazards regression models stratified by sex, recruitment site, and educational level; and adjusted for age, baseline platelet count, diabetes, hypercholesterolemia, hypertriglyceridemia, hypertension, antiplatelet drug use, smoking habit, white blood cells, leisure-time physical activity, body mass index, intake of alcohol, folates, and iron (at baseline, all); and two propensity scores that used 30 baseline variables to estimate the probability of assignment to each of the intervention groups. We used robust standard errors to account for intra-cluster correlations.
MedDiet: Mediterranean diet**.**

SUPPLEMENTAL FIGURES

Supplemental Figure 1. Weighted Kaplan-Meier estimates of the cumulative incidence of thrombocytopenia (A) and thrombocytosis (B) in intervention groups.

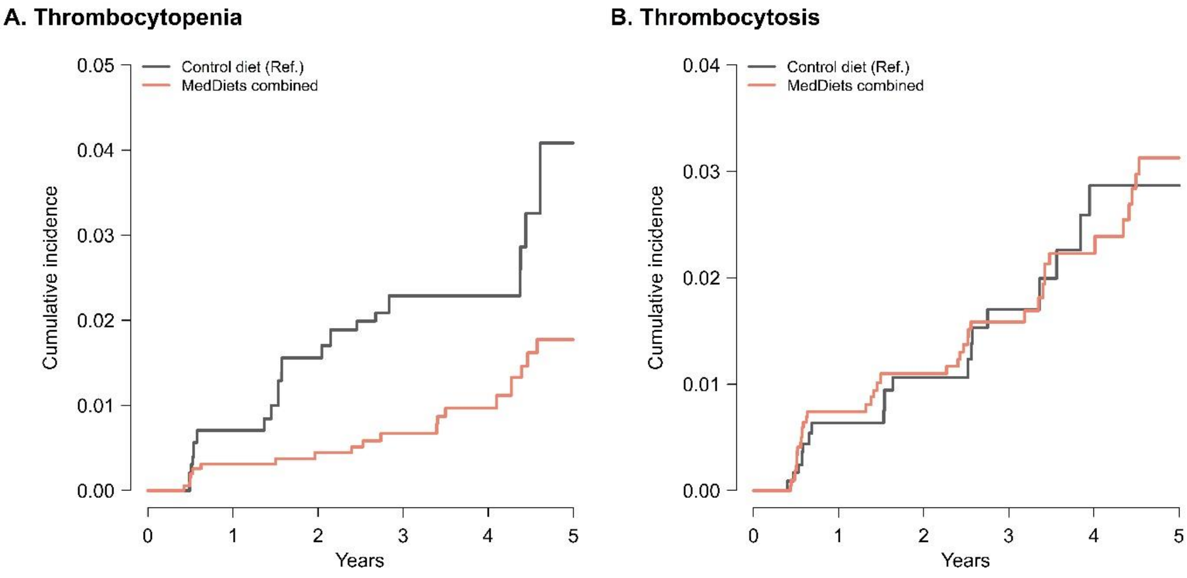

No. at risk (A)

	0	1	2	3	4	5
Control diet	988	891	575	442	291	141
MedDiets	2098	1871	1403	1063	713	367

No. at risk (B)

	0	1	2	3	4	5
Control diet	988	890	576	442	289	143
MedDiets	2098	1862	1402	1065	714	365

Kaplan-Meier curves weighted by inverse probability weighting using a propensity score model of assignment to intervention or control group based on: platelet count at baseline, age, sex, recruitment site, educational level, diabetes, hypercholesterolemia, hypertriglyceridemia, hypertension, smoking habit, leisure-time physical activity, body mass index, white blood cell counts, intake of alcohol, folates, and iron (baseline values, all), and two propensity scores that used 30 baseline variables to estimate the probability of assignment to each of the intervention groups.
MedDiet-EVOO: Mediterranean diet enriched with extra-virgin olive oil; *MedDiet-Nuts*: Mediterranean diet enriched with mixed nuts.

Appendix. List of PREDIMED study collaborators

Hospital Clinic, Institut d'Investigacions Biomèdiques August Pi i Sunyer, Barcelona, Spain: R. Estruch, M. Serra, A. Pérez-Heras, C. Viñas, R. Casas, L. de Santamaría, S. Romero, E. Sacanella, G. Chiva, P. Valderas, S. Arranz, J.M. Baena, M. García, M. Oller, J. Amat, I. Duaso, Y. García, C. Iglesias, C. Simón, L. Quinzavos, L. Parra, M. Liroz, J. Benavent, J. Clos, I. Pla, M. Amorós, M.T. Bonet, M.T. Martin, M.S. Sánchez, J. Altirriba, E. Manzano, A. Altés, M. Cofán, C. Valls-Pedret, A. Sala-Vila, M. Doménech, R. Gilabert, and N. Bargalló.
University of Navarra, Primary Care Centres, Pamplona, Spain: M.Á. Martínez-González, A. Sánchez-Tainta, B. Sanjulián, E. Toledo, M. Bes-Rastrollo, A. Martí, C. Razquin, P. Buil-Cosiales, M. Serrano-Martínez, J. Díez-Espino, A. García-Arellano, I. Zazpe, F.J. Basterra-Gortari, E.H. Martínez-Lapiscina, A. Gea, M. Garcia-Lopez, J.M. Nuñez-Córdoba, N. Ortuño, N. Berrade, V. Extremera-Urabayen, C. Arroyo-Azpa, L García-Pérez, J. Villanueva-Tellería, F. Cortés-Ugalde, T. Sagredo-Arce, Mª D. García de la Noceda-Montoy, Mª D. Vigata-López, Mª T. Arceiz-Campo, A. Urtasun-Samper, Mª V. Gueto-Rubio, and B. Churio-Beraza.
University of Valencia, Valencia, Spain; Universitat Jaume I, Castellon, Spain; and Conselleria de Sanitat, Generalitat Valenciana: D. Corella, P. Carrasco, C. Ortega-Azorín, E.M. Asensio, R. Osma, R. Barragán, F. Francés, M. Guillén, J.I. González, C. Sáiz, O. Portolés, F.J. Giménez, O. Coltell (U. Jaume I), R. Fernández-Carrión, I. González-Monje, L. Quiles, V. Pascual, C. Riera, M.A. Pages, D. Godoy, A. Carratalá-Calvo, S. Sánchez-Navarro, and C. Valero-Barceló.
University Rovira i Virgili, Reus, Spain: J. Salas-Salvadó, M. Bulló, R. González, C. Molina, F. Márquez, N. Babio, M. Sorlí, J. García-Roselló, F. Martin, R. Tort, A. Isach, B. Costa, J.J. Cabré, J. Fernández-Ballart, N. Ibarrola, C. Alegret, P. Martínez, S. Millán, J.L. Piñol, J. Basora, and J.M. Hernández.
Institut Hospital del Mar d'Investigacions Mèdiques, Barcelona, Spain: M. Fitó, M.I. Covas, O. Castañer, S. Tello, J. Vila, H. Schröder, R. De la Torre, D. Muñoz-Aguayo, N. Molina, E. Maestre, A. Rovira, R. Elosua, and M. Farré.
University Hospital of Alava, Vitoria, Spain: F. Arós, I. Salaverría, T. del Hierro, J. Algorta, S. Francisco, A. Alonso-Gómez, J. San-Vicente, E. Sanz, I. Felipe, A. Alonso-Gómez, and A. Loma-Osorio.
University of Málaga, Málaga, Spain: E. Gómez-Gracia, R. Benítez-Pont, M. Bianchi-Alba, J. Fernández-Crehuet Navajas, J. Wärnberg, R. Gómez-Huelgas, J. Martínez-González, V. Velasco-García, J. de Diego-Salas, A. Baca-Osorio, J. Gil-Zarzosa, J.J. Sánchez-Luque, and E. Vargas-López.
Institute of Health Sciences, University of Balearic Islands, and Hospital Son Espases, Palma de Mallorca, Spain: M. Fiol, M. García-Valdueza, M. Moñino, A. Proenza, R. Prieto, G. Frontera, M. Ginard, F. Fiol, A. Jover, and J. García.
Department of Family Medicine, Primary Care Division of Sevilla, Sevilla, Spain: J. Lapetra, M. Leal, E. Martínez, J.M. Santos, M. Ortega-Calvo, P. Román, F.J. García, P. Iglesias, Y. Corchado, E. Mayoral, and C. Lama.

University of Las Palmas de Gran Canaria, Las Palmas, Spain: L. Serra-Majem, J. Álvarez-Pérez, E. Díez-Benítez, I. Bautista-Castaño, I. Maldonado-Díaz, A. Sánchez-Villegas, F. Sarmiendo-de la Fe, C. Simón-García, I. Falcón-Sanabria, B. Macías-Gutiérrez, and A.J. Santana-Santana.
Hospital Universitario de Bellvitge, Hospitalet de Llobregat, Barcelona, Spain: X. Pintó, E. de la Cruz, A. Galera, Y. Soler, F. Trias, I. Sarasa, E. Padres, R. Figueras, X. Solanich, R. Pujol and E. Corbella.
Clinical End Point Committee: F. Arós (chair), M. Aldamiz, A. Alonso-Gómez, J. Berjón, L. Forga, J. Gállego, M. A. García-Layana, A. Larrauri, J. Portu, J. Timiraus, and M. Serrano-Martínez.
